# Influence of product selection criteria on clothing purchase and post-purchase behaviours: A gender and generational comparison

**DOI:** 10.1371/journal.pone.0267783

**Published:** 2022-06-22

**Authors:** Gustavo Barrera-Verdugo, Antonio Villarroel-Villarroel

**Affiliations:** Faculty of Engineering and Business, Universidad de Las Américas, Providencia, Santiago, Chile; Szechenyi Istvan University: Szechenyi Istvan Egyetem, HUNGARY

## Abstract

Purchasing and consumption behaviour is a factor with an important impact on sustainable development. In this regard, the clothing category plays a key role due to the high volume of products that are manufactured in countries with poor environmental and social conditions. While some research has investigated personal, social and cultural conditions that influence these behaviours, little is currently known about the influence of the attributes of sustainable clothing selection on the frequency of sustainable purchase and post-purchase actions in this category. This research seeks to evaluate this effect by comparing the results among genders and age/generation and measuring sustainable consumption using the Young Consumers’ Sustainable Consumption Behaviour method, which has two dimensions: purchase choices and sufficient and frugal consumption. Responses to online surveys of 240 university students in Chile are analysed using descriptive statistics, the Mann-Whitney-Wilcoxon test and ologit regressions. The findings show significant differences between the groups analysed with respect to the influence of attributes for sustainable clothing selection and highlight the negative effect of the search for quality in men and in older people. The evidence highlights the need to inform the population about the characteristics of sustainable clothing that positively affect purchase and post-purchase actions such as buying secondhand clothing, repairing, exchanging and donating clothing. This study also suggests that it is important to strengthen the relationship between sustainability and clothing quality among older generations and men.

## 1. Introduction

The concept of sustainable development was published in 1987 in the report "Our Common Future" [1] and is defined as a form of economic and social development that satisfies people’s present needs without harming the ability of future generations to satisfy their own needs. In turn, in line with sustainable development, the United Nations (n.d.) [2] states that sustainable production and consumption implies the efficient use of resources and energy, the construction of environmentally friendly infrastructure and the creation of environmentally friendly jobs with fair pay and good working conditions. From the perspective of consumption, these approaches condition the preference, purchase, use and disposal of products in accordance with the care of natural resources, the use of efficient energy and adequate working conditions. Another concept that favours sustainable production and consumption is fair trade, defined by the Word Fair Trade Organisation [3] as follows: "A trading system based on dialogue, transparency and respect, which seeks greater equity in international trade with special attention to social and environmental criteria. It contributes to sustainable development by offering better trading conditions and securing the rights of disadvantaged producers and workers, especially in the South" (p.6).

Along these lines, in 2015, the United Nations defined the 2030 Agenda for Sustainable Development, an initiative that incorporates seventeen goals that have been included in the report "The 2030 Agenda and the Sustainable Development Goals. An opportunity for Latin America and the Caribbean", published by CEPAL [4]. Goal twelve demands ensuring sustainable consumption and production patterns. Sustainable consumption is defined as “the use of goods and services that respond to basic needs and bring a better quality of life, while minimising the use of natural resources, toxic materials and emissions of waste and pollutants over the life cycle, so as not to jeopardize the needs of future generations” [5].

Due to the recognised importance of sustainable clothing consumption, a significant amount of research has attempted to understand the consumption habits of population groups in different countries in the last ten years [6, 7] and to recognise which factors are related to or influence sustainable clothing consumption behaviours [8, 9]. In the measurement of sustainable consumption behaviours, different scales have been developed to determine their magnitude [10, 11]. Among these methodologies, the Young Consumers’ Sustainable Consumption Behaviour (YCSCB) scale [12] is a recent measurement tool that is focused on the clothing and food category, and is accepted and valued. YCSCB presents two measurement factors in the clothing category: the first is called sufficient and frugal consumption, which is related to exchanging and borrowing clothes, buying second-hand clothes and repairing clothes, and the second is called purchase choices, which are associated with the selection of products for environmental care, social responsibility or high quality.

Thus, the two YCSCB dimensions cover sustainable consumption in a global way, focusing on different and complementary areas: purchase choices are related to sustainable attributes that are valued in the choice of clothing, while sufficient and frugal consumption evaluates sustainable behaviours integrated with self-production, purchase and post-purchase, such as the acquisition of secondhand clothing or self-production, repair, transformation, donation or exchange of clothing. It is also possible to argue that the purchase choices factor is linked to attribute-based product selection theory, which proposes that consumers use their preferences for products based on their attributes in terms of their functional and psychological benefits or risks (consequences) to achieve their underlying values [13]; in contrast, the sufficient and frugal consumption factor is linked to the measurement of observable behaviours, which are subsequent to preference and purchase intention, considering the response hierarchy models in the field of consumer behaviour as a basis that order consumption hierarchies into three stages: cognitive, affective and behavioural [14, 15]. In addition, Sufficient and frugal consumption incorporates the measurement of fashion clothing disposal behaviour, a type of post-purchase behaviour that is defined as the act of getting rid of something, in other words, the end of the clothing’s life with the current owner, encompassing whether the clothing is discarded as scrap or given for recycling or reuse [16].

As mentioned, these two YCSCB factors address different aspects, and in view of their discrepancies, the following questions arise: Does people’s evaluation of attributes for selecting clothing included in purchase choices impact the post-purchase actions embedded in sufficient and frugal consumption? Does the impact differ according to people’s gender and age group? These questions are important because to date, there is little information regarding the effect of sustainable attributes in clothing selection on the frequency of sustainable purchase and post-purchase actions among different segments of the population. Most of the research published to date that has attempted to recognise determinants of sustainable purchase and post-purchase behaviour has analysed personal demographic or psychological characteristics [17, 18], the influence of the social environment [19, 20] and the impact of cultural conditions [21, 22] on behaviours such as the purchase of second-hand clothing or post-purchase behaviours such as fashion clothing disposal behaviour.

Consequently, a knowledge gap that implies a research problem is recognised. It is concerning the lack of understanding of which attributes of sustainable clothing influence the frequency of behaviours in the purchase and post-purchase stages that benefit environmental care and social wellbeing. Precisely, the objective of this research is to analyse the influence of the attributes for sustainable clothing selection incorporated in the purchase choices dimension on sustainable purchase and post-purchase behaviours assessed in sufficient and frugal consumption, recognising differences among genders and age/generation. This research uses the YCSCB measurement method because it has been validated and incorporated in studies about sustainable consumption [12, 23, 24]. The YCSCB scale is similar to other metrics developed to assess sustainable consumption in different segments of populations [8, 25]; consequently, we consider YCSCB to be a valid instrument to measure sustainable consumption of clothing in this research.

Searching new evidence to answer these questions and achieve the objective of this research is important, as it would help understand which attributes of clothing selection are associated with subsequent behaviours that reduce the use of natural resources and the generation of pollutants from clothing waste. This information could be used by governmental and nongovernmental organisations to promote the valuation of some attributes with a favourable impact on positive purchasing and post-purchase behaviours from a sustainability perspective, such as the purchase of second-hand clothing and the repair, exchange and donation of clothing. Complementarily, knowing differences by gender and age enhances the usefulness of the findings due to published evidence that distinguishes sustainable consumption behaviours by gender and age generation [25, 26] and because segmenting the results by these demographic variables facilitates the development of targeted strategies in the marketing areas of companies. Organisations could capture the preference of demographic segments, by gender or age, combining the attributes valued by these groups for product selection with benefits or sales promotions that facilitate the purchase of second-hand clothing, repair, donation, exchange or reuse of garments.

## 2. Theoretical frame

### 2.1. Sustainable consumption

Sustainable consumption is defined as the consumption of goods and the environmental costs and economic inequality associated with the modern consumerist lifestyle. In addressing environmental issues, policy-makers should not only focus their attention on the production side of industrial companies but also encourage responsible consumer consumption behaviour [27]. Similarly, Wang and Ward [28] stated that sustainable consumption consists of the consumption of goods and services that are produced using techniques and materials that cause minimal damage to the environment and that satisfy the basic needs of all human beings in a socially equitable manner. Complementarily, sustainable consumption can be defined as "the use of goods and services that satisfy basic needs and provide a better quality of life by minimising the use of natural resources, toxic materials and waste and pollutants throughout their life cycle, so as not to threaten the needs of future generations" [5]. These definitions make it clear that sustainable consumption incorporates both the production conditions of products, including the natural and human resources used in their manufacture and sale, as well as purchase and post-purchase behaviours related to the acquisition of second-hand products, exchange, reuse, donation or transformation; these behaviours are carried out within the life cycle of products and contribute to a lower use of natural resources, toxic materials and waste by avoiding or postponing the purchase of new products.

### 2.2. Sustainable clothing attributes

Attribute-based product selection theory proposes that consumers prefer products according to their attributes in terms of their functional and psychological benefits or risks (consequences) [13]. This theory also assumes that consumers’ decision-making is a form of problem solving (rather than cognitive rationalisation) in the sense that consumers will resolve their problems by taking various actions to maximise benefits and avoid negative outcomes [29]. The attributes that are considered in product selection are defined as properties or characteristics of a product that are intrinsic to it, or in other words, that are linked to it and are concrete, observable, objectively measurable and relevant for choosing between alternatives [30]. Keller [31] argued that product attributes refer to a descriptive characteristic of a brand and the components of the product that customers will look for. Complementarily, Keller [32] stated that direct product-related attributes are the materials necessary for the product to function, usually linked to its physical composition, and that nonproduct-related attributes are external aspects of a product, such as information about the product’s price, packaging and design, including people, groups or celebrities who use the product.

In relation to attributes relevant to the selection of sustainable apparel, Patnaik et al. [33] posited that sustainable fashion products are manufactured organically and produce less wastewater and harmful chemicals; in this regard, an increasing number of apparel companies are creating quality garments and building successful brands while developing sustainable practices, such as the use of recycled and organic materials. Similarly, Rausch et al. [34] pointed out that the most important attributes of sustainable clothing are the durability of the garment, fair wages and working conditions, as well as an environmentally friendly production process. In YCSCB, Fischer et al. [12] proposed that the attributes for the selection of sustainable apparel are: production in countries with good working conditions, compliance with fair trade in the sale of clothing, the incorporation of organic production, the absence of pollutants in the clothing, and the high quality and durability of the clothing.

### 2.3. Clothing purchasing and post-purchase behaviours

Response hierarchy models published in the 20th century [35, 36], which describe the stages consumers go through in the buying process, propose three general stages. The first stage is called "cognitive" and is related to customers’ awareness and knowledge of a product and brand. The second stage is called "affective" and is associated with the emotions that products or brands generate in customers. The third stage is called "behavioural" and is related to behaviours that can be observed and recorded, such as buying, consuming and discarding products. Examples of the use of these three stages are the AIDA model [37], which determines the attention stage in the cognitive phase, interest and desire in the affective phase and action in the behavioural phase; the Hierarchy of Effects model [38], which recognises awareness and knowledge of the product in the cognitive phase, taste and preference for the product in the affective phase, and the purchase of the product in the behavioural stage. In the last phase, which is the behavioural stage, it is possible to recognise various behavioural indicators, such as the brand purchased, the sales channel used in the purchase, the method of payment used in the purchase, and the frequency and volume of purchase. Some models also include post-purchase as a later stage. In this respect, Kotler and Turner [39] explain that customer satisfaction is the consequence of the customer’s experience during the distinct phases of the purchase process, which includes the stages of awakening of the need, information search, evaluation of alternatives, purchase decision and post-purchase behaviour.

In the area of sustainable clothing purchasing behaviour, one widely studied conduct is the purchase of second-hand clothing, since the repurchase and reuse of clothing reduces the use of natural and human resources for the manufacture and distribution of new clothing. Regarding post-purchase behaviours, Bianchi and Birtwistle and Laitala [6, 16] stated that in the field of clothing, post-purchase includes actions such as the use, care, reuse, destruction or disposal of garments. Reuse of clothing may involve giving or transferring garments to friends or family, while donated clothing may be exported and sold as second-hand clothing to consumers, especially in third world countries [40]. Recycled clothing can also be converted into new products [41].

A concept related to post-purchase behaviours is the term "disposal of unused product". In the case of clothing, Chun [42] defined this behaviour as “discontinued wear and disposal of a clothing item by giving it to others, throwing it away, using it for another purpose than wearing apparel, or selling it at used clothing stores or garage sale” (p. 7). More recently, Laitala [16] defined this disposal as "the act of getting rid of something, i.e., the end-of-life stage of the clothing with the present owner, regardless of whether the clothing is discarded of as waste or delivered to recycling or reuse" (p. 444). In this regard, there are several ways in which consumers can dispose of clothing: donating it to charity, giving it to family or friends, reselling it, or throwing it away. Donating it to charity and giving it to family or friends are common methods of reusing clothing [43, 44].

The observable post-purchase behaviour of clothing is highly relevant from the perspective of environmental and social sustainability, as it affects the volume of new clothing that is manufactured and distributed using natural and human resources. The clothing sector is characterised by the generation of a high volume of waste that can be minimised with actions such as the reuse of garments, donation, exchange or recycling. Clothing production has increased significantly in the last decade, and the number of times consumers wear clothes has decreased by one-third since the beginning of the century. Complementarily, clothing sales will increase to 160 million tonnes by 2050 [45], and global consumption of clothing will reach approximately 102 million tonnes by 2030 [46]. Furthermore, many of the companies in the textile and clothing industry implement production and sales strategies with a focus on massification and low prices; these strategies increasingly encourage consumers to buy, which has a negative impact on the environment and society [47].

To date, some of the research has focused on studying the habits and trends in population groups in different countries regarding secondhand clothing purchases [48, 49] and fashion clothing disposal behaviour [50, 51]. Other research has searched for factors that affect these behaviours. Gopalakrishnan and Matthews [52] argued that cheaper prices, the excitement of finding great deals, brand value and variety are the main reasons for shopping at second-hand shops. Arangdad et al. [17] stated that the physical condition of clothing and lack of awareness are the most prominent reasons preventing consumers from recycling more textile products. Pingki and Kuntala [53] recognised that creating more infrastructural facilities for recycling clothing, expanding environmental and social awareness, and implementing corporate social responsibility (CSR) activities should positively affect sustainable clothing disposal.

Regarding clothing attributes that affect purchase and post-purchase behaviour, Paço et al. [26] have argued that the main barriers to buying secondhand clothing are the perceived lower cleanliness of the garment and the fact that these clothes have already been previously used by other people. McQueen et al. [54] stated that people are less likely to donate, give away or sell malodorous clothing and are more likely to dispose of malodorous garments directly in the rubbish. Degenstein et al. [55] indicated that the severity of damage to clothing caused by its use plays a key role in how people decide to dispose of their unwanted garments and that the quality and type of garment predict the disposal method and end-of-life extension strategies. Similarly, Laitala and Klepp [56] argued that uniqueness and style are more important for those who buy second-hand clothes and that barriers to buying used clothes are lack of hygiene, health and intimacy.

### 2.4. Clothing consumption in Chile

Chile is one of the countries in Latin America with the highest per capita clothing consumption. In the last 5 years, people in Chile have increased their clothing consumption by 80%, going from 13 to 50 new garments per year on average [57]. The high access to credit in the population [58] and the fast fashion marketing strategies implemented by mass retail that are based on discounts and the distribution of credit cards for payment in instalments [59, 60] allow the middle socioeconomic groups to purchase clothing from fast fashion brands such as Zara or H & M.

The arrival of used clothing from the United States to Chile began in 1975 under sanitary and customs regulations [61]. Thus, the purchase of secondhand clothing is accessible because in several cities, there are stores that sell used clothes. “American clothing" or "European clothing" stores that sell used clothing have been available in the country for decades [62]. The consumption of second-hand clothing is transversal among Chile’s social classes, it takes place in street sells with low prices and in stores that offer exclusivity, clothes for important occasions, costumes and very large or small special sizes. Besides, the brands positioned in the local second-hand offer: online sales channels for wholesalers, garment exchange guarantees, and application of the "recycled clothing" concept linked to environmental care and social responsibility [61]. In addition, some large department stores are now accepting used clothing as part payment for new clothing, this commercial strategy is mainly focused on the younger segments of the market.

Moreover, Chile is a country with the highest internet penetration, with more than 14 million people connected, representing 76% of the population [63], and more than 16 million users of social media [64]. This broad digital coverage facilitates the population’s access to online shopping for clothing, both new and second-hand, and facilitates the population’s access to clothing exchange; in this sense, online platforms for buying and exchanging used clothing are currently prospering in the country [65].

With respect to the information provided to consumers, the garments include labels that identify the country of manufacture of the garment and its materials, this information have been legally defined in Chile [66]. Consequently, it is easy for a person to recognise whether the garment is manufactured in an Asian country and whether it is made of cotton or a synthetic material such as polyester. In addition, due to the high internet and social media coverage, the population has access to multiple online sources of information that facilitate the knowledge of the negative environmental and social conditions of the countries that contribute to fast fashion.

## 3. Hypothesis

Regarding of the influence of valuing sustainable attributes in clothing on sustainable purchase and post-purchase behaviours, no recent evidence has been recognised associating these selection attributes with the frequency of these post-purchase behaviours. However, because the influence of other types of non-sustainability-related attributes on second-hand purchasing or clothing disposal behaviour has been supported, such as the smell and cleanliness of clothing [56, 54], the perception that clothing is out of fashion [41] and high brand recognition of the product due to its authenticity and quality [67], this research suggests that valuing conditions such as organic production, fair-trade manufacturing, absence of polluting materials and quality should affect the frequency of clothing purchase and post-purchase actions that are important for social and environmental sustainability, such as exchanging, reusing, repairing, donating, disposing of clothes and buying second-hand clothes. This approach is also justified by the phases exposed in the response hierarchy models [38, 36] which support that the preference or desire for a product precedes subsequent behaviors such as its purchase and consumption. Under this theoretical framework, the valuation of attributes for clothing preference or selection should impact subsequent purchase and post-purchase behaviours. Consequently, the following research hypothesis is proposed:

- Hypothesis 1: The valuation of sustainable attributes of clothing affects the frequency of sustainable purchase and post-purchase consumption behaviours.

Complementarily, previous research has recognised discrepancies in sustainable consumption by gender. Isenhour and Ardenfors [68] concluded that women are more likely than men to consume sustainably, considering activities such as buying organic and fair-trade products, reducing travel, consuming organic food and recycling; according to their research, women express more interest in sustainable living and spend more time seeking information and alternatives on sustainable consumption. Bulut et al. [25] noted that women show a higher level of sustainable consumption behaviour both in general behaviour and in the tendency to reuse products. More recently, Nencková et al. [69] supported that men are significantly more likely to throw away their clothes if they do not use them, and Kopplin and Rösch [70] have argued that women are willing to buy sustainable clothes only due to their concern for the environment. Previous research has argued a higher tendency towards sustainable consumption in women with few exceptions; for example, Lang et al. [71] stated that sensitivity to fashion trends, higher income and female gender are positively correlated with frequent clothing disposal. Supported by the gender differences evidenced in previous publications, this research proposes that the influence of attributes for sustainable product selection on the frequency of purchase and post-purchase behaviour should be different between men and women, and consequently, the following research hypothesis is proposed.

- Hypothesis 2: The valuation of sustainable attributes of clothing differentially affects the frequency of sustainable purchase and post-purchase consumption behaviours between men and women.

Additionally, generational differences in sustainable consumption have been argued. Bulut et al. [25] noted that older consumers of the Baby Boom generation have the highest level of unnecessary consumption of products, while younger consumers of generation Z have the lowest level of unnecessary consumption. Similarly, several studies published in the last 5 years have recognised an important trend of the centennial or "Z" generation towards environmental and social concern [72–74], which is also expressed in an important propensity towards sustainable clothing consumption behaviours [75, 76]. Supporting generational differences in sustainable consumption, Paço et al. [26] stated that young consumers are more likely to buy textile products in second-hand markets due to environmental reasons, lack of money or the search for vintage fashion. Additionally, Liang and Xu [77] analysed four generational cohorts in China, differentiating between people born in the 1960s, 1970s, 1980s and 1990s, observing that post-70s consumers avoided buying secondhand clothing, while younger generations perceived higher values and had higher purchase intentions for secondhand clothing than their older counterparts. On the basis of such generational differences, this research hypothesises that the influence of sustainable clothing selection attributes on these subsequent behaviours should also be different among the centennial generation:

- Hypothesis 3: The valuation of sustainable attributes of clothing differentially affects the frequency of sustainable purchase and post-purchase behaviours between the centennial generation and older people.

## 4. Methodology and methods

### 4.1. Measurement

Online surveys were distributed through the Survey Monkey platform. Respondents answered the online survey independently without the presence of an evaluator. The measurement in the survey is based on the Young Consumers’ Sustainable Consumption Behaviour (YCSCB) scale published by Fischer et al. [12]. They argued that YCSCB is based on the Sustainable Consumption Behaviour Cube (SCB-Cube) proposed by Geiger et al. [10]. The SCB-Cube provides an integrative conceptual framework that includes three domains of sustainable consumption; the first is related to the different areas of consumption (food, housing, mobility, clothing, etc.), the second is associated with the phases of consumption (acquisition, use and disposal of consumer goods), and the third domain involves the selection of consumption impacts in two dimensions of sustainability (ecological and socioeconomic).

Fischer et al. [12] applied this framework to develop the YCSCB in the clothing categories, addressing the stages acquisition, usage, and disposal of consumer goods. They recognised, after conducting a qualitative and quantitative study, that the sentences “I avoid buying clothing items that originate in countries with poor working conditions” and “I choose clothing items from fair trade production” are related to the socioeconomic dimension in the acquisition stage, and the other phrases, including "I choose high-quality and long-lasting clothing items", are linked to the ecological dimension in the acquisition stage.

The choice of high-quality and long-lasting apparel can be associated with the concept of slow fashion. This is a socially conscious movement that shifts the mindset of consumers from quantity to quality, encouraging people to purchase high-quality items less frequently [78]. Hall [79] argued that slow fashion involves longer production times, the use of local materials, and a focus on quality and sustainability. In other words, slow fashion implies a longer useful life of the garment from manufacture to disposal, thus reducing the amount of clothing that needs to be manufactured and distributed and reducing the resources used (materials, transportation, infrastructure, etc.), affecting sustainability from an ecological perspective.

The YCSCB scale is similar to other metrics developed to assess sustainable consumption. Razzaq et al. [8] used the statements: “I will buy clothing that is made with recycled content”, “I will buy clothing that can be disposed of in an environmentally friendly manner”, “I will buy clothing that is safe for the environment”, “I will limit my use of that clothing that is made of or uses scarce resources”, “I will not buy new clothing items if I already have previous dresses in a usable state”, and “I will buy clothing that is produced in an environmentally friendly manner”. Complementarily, Bulut et al. [25] evaluated sustainable consumption behaviours among genders and generations using a four-dimensional sustainable consumption behaviour scale that includes coherent and similar statements to YCSCB. Consequently, we consider YCSCB to be a valid instrument to measure sustainable consumption of clothing in this research, such that it has incorporated sets of validated statements for measuring sustainable consumption that have also been used in previous studies related to this issue [23, 24].

As noted previously, YCSCB incorporates two factors: purchase choices, which are associated with the importance of sustainable attributes in product selection, and sufficient and frugal consumption, which measures purchase and post-purchase behaviours that affect environmental and social sustainability. The YCSCB items were adapted to the Chilean context, translated from English into Spanish, and then translated back into English for presentation in this research. Before final distribution, the survey was answered by a small sample of twenty students to check the understanding of the statements and make corrections. A frequency scale was used in the measurement with the following levels associated with numbers: never (1), rarely (2), occasionally (3), sometimes (4), frequently (5), very frequently (6), and always (7). Seven levels were selected in this scale instead of five to allow the selection of a larger number of frequency options for the respondents. The 7-level frequency scale has been used for decades to assess the frequency of observable behaviours or the frequency of preferences and intentions in research [80–82].

### 4.2. Sample

A total of 240 students from the Faculty of Engineering and Business at Universidad de Las Américas in Chile completed the online survey in full, 105 men, 133 women and two persons who did not respond or indicated another gender. The average age of the students was 29.41 years, and the standard deviation of their age was 9.45 years. The classification of the centennial generation considers the assessed persons who were born since 1996, according to the classification defined by Lanier [83].

The students surveyed are from the lower-middle, middle and upper-middle socioeconomic classes of Chile, which represent approximately 60% of the country’s population [84]. Students are enrolled in a private university; in addition, they pay a college fee and have access to the internet because they had to attend online classes since the beginning of the COVID-19 pandemic. Consequently, students have access to buy clothes in mass retail, in physical stores or through e-commerce, as well as to buy new and used clothes on social media.

Most of the students who responded to the survey were enrolled in Commercial Engineering and Business Administration Engineering. The survey was sent to approximately 1,000 students from March 2020 to October 2021, representing a response rate of nearly 24%. The 240 surveys selected for analysis in this research are those that were fully responded. All surveys included a request for informed consent that was defined by the Ethics Committee of Universidad de Las Américas (ethic committee code CEC_FP_2019021). Only responses of students who accepted informed consent were considered. Table 1 details the characteristics of the student sample.

**Table 1:** Description of the sample.

	Average age	Standard deviation of age	Commercial Engineering	Business Administration Engineering	Industrial Engineering	Other Careers	Total
**Total centennials**	21.24	1.88	68	19	8	20	**115**
Centennials men	21.32	1.83	32	6	5	3	**46**
Centennials women	21.19	1.93	36	12	3	17	**68**
Centennials other-gender	19.00	0.00	0	1	0	0	**1**
**Total Older**	36.94	7.05	52	42	23	8	**125**
Older men	37.17	7.60	22	16	19	2	**59**
Older women	36.89	6.49	29	26	4	6	**65**
Older other-gender	26.00	0.00	1	0	0	0	**1**
**Total**	**29.41**	**9.45**	**120**	**61**	**31**	**28**	**240**

### 4.3. Analysis

Statistical analysis of the data was performed with the statistical package STATA version 16. First, a comparative analysis of the observed variables by gender and age generation was conducted using the arithmetic mean and an evaluation of differences between response distributions through the Mann-Whitney-Wilcoxon test. This test is a nonparametric evaluation whose null hypothesis is based on equal distributions of both populations [85] and that involves the comparison of the mode representing the 50% percentile of the data distribution. Obtaining a P value < 0.05 supports statistically significant differences. The Mann–Whitney test is used for comparison between two groups having quantitative variables that are not normally distributed [86]; likewise, it is a robust competitor of the t-test in the univariate setting [87].

This research seeks to evaluate the criteria influence for selecting sustainable clothing on post-purchase actions. Therefore, a logistic regression analysis was performed for ordinal variables, considering the product selection attributes incorporated in the purchase choices factor as independent variables and the purchase and post-purchase actions of clothing, associated with the sufficient and frugal consumption factor, as dependent variables. Logistic regression is typically used to evaluate the influence of a set of variables on the selection probability of a certain alternative, especially for factors that affect a given decision but cannot be directly observed [88]. Ordinal logistic regression is a regression analysis used to find and describe the relationship between ordinal scale response variables with more than two categories and a set of continuous or categorical predictor variables [89]. It is a technical statistical technique capable of processing data on an ordinal scale in situations involving several factors that can influence the outcome [90]. The overall fit of the regressions is assessed by the Chi2 test, and the significance of the regression coefficients is assessed by hypothesis testing based on the parameter P>|z|.

Moreover, the Mann-Whitney-Wilcoxon test and logistic regressions were selected because the responses obtained for the observed variables did not show a normal distribution, considering a confidence level of 99% or 95%, except for the responses obtained for the statement "I choose high quality and long-lasting clothing items". The normal distribution of the variables was evaluated with the Shapiro-Wilks test, and the results showed p-values of less than 0.05 or 0.01, which represents the absence of a normal distribution; only the measurement of the quality attribute showed a higher p-value (0.05).

## 5. Results

### 5.1. Comparison of arithmetical means by groups

Table 2 shows the arithmetic means obtained for each group evaluated. These arithmetic means suggest that the female group and centennials express a higher frequency of behaviours associated with the sufficient and frugal consumption factor. The Man-U Wilcox test also supports these differences in the distribution of response frequencies between men and women and between centennials and older people. Although the arithmetic means obtained show a low frequency of behaviours in general terms, women show higher magnitudes in the statements "I buy second-hand clothing" (p<0.01), "I wear patched and mended clothing" (p<0.01) and "Instead of buying a new piece of clothing for a special occasion, I borrow something" (p<0.01). For the centennial generation, the magnitudes are higher for the statements "I buy second-hand clothing" (p<0.01), "I wear patched and mended clothing" (p<0.05), "instead of buying a new piece of clothing for a special occasion, I borrow something" (p<0.10), and "I look for other possible uses of unwanted clothing items" (p<0.05).

**Table 2:** Comparison of central tendency by group.

**Sufficient and frugal consumption**	**Mean Women**	**Mean Men**	**Prob > |z| Mann-U Gender**	**Mean Centennials**	**Mean Olders**	**Prob > |z| Mann-U Generation**
I buy second-hand clothing.	3.23	2.26	**0.00**	3.32	2.34	**0.00**
I wear patched and mended clothing.	3.53	2.80	**0.00**	3.49	2.96	**0.01**
I give away or swap unwanted clothing items that I no longer wear.	4.57	4.33	0.28	4.25	4.64	0.12
Instead of buying a new piece of clothing for a special occasion, I borrow something.	2.53	1.87	**0.00**	2.40	2.09	**0.05**
I look for other possible uses of unwanted clothing items.	3.84	3.42	0.11	3.96	3.35	**0.02**
I air my clothing items properly before deciding whether they need washing.	3.66	3.58	0.83	3.69	3.54	0.49
I throw away clothing items that I no longer wear.	3.38	3.44	0.92	3.22	3.61	0.29
**Purchase choices**	**Mean Women**	**Mean Men**	**Prob > |z| Mann-U Gender**	**Mean Centennials**	**Mean Olders**	**Prob > |z| Mann-U Generation**
I avoid buying clothing items that originate in countries with poor working conditions.	2.68	2.66	0.86	2.79	2.54	**0.09**
I choose clothing items from fair-trade production.	3.54	3.38	0.42	3.51	3.46	0.41
I choose clothing items from organic production (e.g., made from organic cotton).	3.38	3.23	0.47	3.43	3.18	0.14
I select clothing that includes labels guaranteeing the absence of chemical pollutants.	2.97	3.22	0.32	3.20	2.95	0.10
I choose high quality and long-lasting clothing items.	4.36	4.86	**0.02**	4.28	4.85	**0.01**

Note: A *p* value <0.01 represents a significant difference with 99% confidence, a *p v*alue <0.05 represents a significant difference with 95% confidence, and a *p* value <0.10 represents a significant difference with 90% confidence.

In the case of the purchase choices factor, significant differences with 95% (p<0.05) confidence were only recognised in the statement "I choose high quality and long-lasting clothing items". The arithmetic mean of the importance of this selection criterion is higher for men and older people; therefore, these results suggest that in these groups, quality is a more relevant attribute. Additionally, the centennial generation shows a higher concern than older people in avoiding clothes items that originate in countries with poor working conditions, and the difference in distribution in this statement is significant only at 90% confidence (p<0.10) according to the Man-U Wilcox test.

### 5.2. Ologit regressions by gender

Table 3 presents the ologit regressions in the women’s group, including the statements of sufficient and frugal consumption as dependent variables. The Chi2 test (prob > chi2) shows that the regressions with the dependent variables "I buy secondhand clothing", "I give away or swap unwanted clothing items that I no longer wear", "instead of buying a new piece of clothing for a special occasion, I borrow something", "I look for other possible uses of unwanted clothing items", and "I air my clothing items properly before deciding whether they need washing" have a valid goodness of fit considering a 99% (p <0.01), 95% (p <0.05) or 90% (p <0.10) confidence level. In contrast, the regressions associated with the action "I wear patched and mended clothing" and "I throw away clothing items that I no longer wear" as the dependent variable did not obtain good goodness of fit. In the regressions with adequate goodness-of-fit, significant regression coefficients were found at 99% (p<0.01), 95% (p<0.05) or 90% (p<0.10) confidence. In particular, the statement "I avoid buying clothing items that originate in countries with poor working conditions" shows a high positive regression coefficient (β = 0.43, P>|z| = 0.00) in the regression with the dependent variable "I buy secondhand clothing"; the statement "I choose clothing items from fair trade production". (β = 0.46, P>|z| = 0.00) in the regression with the dependent variable "I give away or swap unwanted clothing items that I no longer wear" also stands out for its high positive and significant coefficient. Mainly, it is recognised that among women, the concern to avoid garments that originate in countries with poor working conditions has a positive effect on the behaviour of buying second-hand clothes, borrowing clothes, finding new uses for clothing, and airing clothes. In a complementary way, it can be seen that only quality has a negative effect on the purchase of second-hand clothes (β = -0.17, P>|z| = 0.08) and has a positive effect on the donation or exchange of clothes (β = 0.18, P>|z| = 0.07).

**Table 3:** Logistic regression of women.

	I buy second-hand clothing		I wear patched and mended clothing		I give away or swap unwanted clothing items that I no longer wear		Instead of buying a new piece of clothing for a special occasion, I borrow something		I look for other possible uses of unwanted clothing items		I air my clothing items before deciding whether they need washing		I throw away clothing items that I no longer wear	
	β	P>|z|	β	P>|z|	β	P>|z|	β	P>|z|	β	P>|z|	β	P>|z|	β	P>|z|
I avoid buying clothing items that originate in countries with poor working conditions	**0.43**	**0.00**	**0.27**	**0.02**	-0.10	0.36	**0.31**	**0.01**	**0.23**	**0.04**	**0.16**	**0.09**	0.15	0.21
I choose clothing items from fair-trade production	-0.03	0.82	0.00	0.99	**0.46**	**0.00**	-0.08	0.52	0.01	0.92	0.19	0.10	-0.13	0.24
I choose clothing items from organic production (e.g., made from organic cotton)	0.11	0.42	0.07	0.59	-0.02	0.85	0.17	0.19	0.12	0.35	-0.08	0.50	0.05	0.67
I select clothing that includes labels guaranteeing the absence of chemical pollutants	-0.20	0.12	-0.05	0.70	-0.06	0.61	-0.10	0.44	0.10	0.43	0.19	0.13	-0.16	0.17
I choose high quality and long-lasting clothing items	**-0.17**	**0.08**	0.02	0.84	**0.18**	**0.07**	-0.06	0.55	-0.07	0.45	0.03	0.76	0.13	0.17
Observations	133		133		133		133		133		133		133	
Prob > chi2	0.00		0.10		0.00		0.07		0.02		0.00		0.37	
Pseudo R2	0.04		0.02		0.05		0.02		0.03		0.04		0.01	

Table 4 presents the ologit regressions in the men’s group, also incorporating the sufficient and frugal consumption statements as dependent variables. In this case, the results show that all the ologit regressions, except the regression with the dependent variable "I throw away clothing items that I no longer wear", obtained a good goodness of fit (Chi2 test with p<0.01). The main difference with respect to women is that significant negative regression coefficients were obtained, at 99% or 95% confidence, particularly these associated with the statement "I choose high quality and long-lasting clothing items". This result shows that, in the case of men, the search for quality negatively affects purchase and post-purchase behaviours that benefit environmental care and social well-being, particularly the actions "I buy second-hand clothes" (β = -0.35, P>|z| = 0.00), "I wear patched and mended clothing" (β = -0.33, P>|z| = 0.00), "instead of buying a new piece of clothing for a special occasion, I borrow something" (β = -0.28, P>|z| = 0.03) and "I look for other possible uses of unwanted clothing items" (β = - 0.41, P>|z| = 0.00). In contrast, the results show that the preference for organic production positively affects the purchase of secondhand clothes (β = 0.37, P>|z| = 0.05), patching or mending clothes to continue using them (β = 0.47, P>|z| = 0.00), and airing clothes before deciding to wash them (β = 0.51, P>|z| = 0.00). Complementarily, it is recognised that avoiding clothing items that originate in countries with poor working conditions has a positive effect on the actions "I buy second-hand clothing" (β = 0.28, P>|z| = 0.08) and “I throw away clothing items that I no longer wear” (β = 0.29, P>|z| = 0.05), and that selecting clothes that are produced under fair trade has a positive effect on post-purchase action "I air my clothing items properly before deciding whether they need washing" (β = 0.28, P>|z| = 0.03) and a negative effect on post-purchase action “I throw away clothing items that I no longer wear” (β = -0.37, P>|z| = 0.01).

**Table 4:** Logistic regression of men.

	I buy second-hand clothing		I wear patched and mended clothing		I give away or swap unwanted clothing items that I no longer wear		Instead of buying a new piece of clothing for a special occasion, I borrow something		I look for other possible uses of unwanted clothing items		I air my clothing items before deciding whether they need washing		I throw away clothing items that I no longer wear	
	β	P>|z|	Β	P>|z|	β	β	P>|z|	β	P>|z|	P>|z|	β	P>|z|	β	P>|z|
I avoid buying clothing items that originate in countries with poor working conditions	**0.28**	**0.08**	0.06	0.65	0.22	0.13	0.26	0.10	0.16	0.27	0.00	0.99	**0.29**	**0.05**
I choose clothing items from fair-trade production	0.16	0.30	0.13	0.37	0.06	0.66	0.23	0.13	0.11	0.45	**0.28**	**0.03**	**-0.37**	**0.01**
I choose clothing items from organic production (e.g., made from organic cotton)	**0.37**	**0.05**	**0.47**	**0.00**	0.03	0.84	-0.08	0.67	0.25	0.13	**0.51**	**0.00**	0.27	0.12
I select clothing that includes labels guaranteeing the absence of chemical pollutants	-0.11	0.53	-0.00	0.99	0.16	0.30	**0.35**	**0.04**	0.13	0.41	-0.20	0.17	-0.16	0.32
I choose high quality and long-lasting clothing items	**-0.35**	**0.00**	**-0.33**	**0.00**	0.11	0.29	**-0.28**	**0.03**	**-0.41**	**0.00**	0.14	0.17	0.01	0.91
Observations	105		105		105		105		105		105		105	
Prob > chi2	0.00		0.00		0.00		0.00		0.00		0.00		0.13	
Pseudo R2	0.07		0.08		0.05		0.09		0.06		0.08		0.02	

### 5.3. Ologit regressions by age generation

Table 5 shows the results linked to the centennial group. According to the Chi2 test, the regressions with the dependent variables "I buy second-hand clothing", "I wear patched and mended clothing", "I give away or swap unwanted clothing items that I no longer wear" and "I air my clothing items properly before deciding whether they need washing" obtained adequate goodness of fit at 99% (p <0.01), 95% (p <0.05) or 90% (p <0.10) confidence. The significant regression coefficients are mostly associated with the purchase of secondhand clothing as the dependent variable. In this regression, the statements "I avoid buying clothing items that originate in countries with poor working conditions" (β = 0.39, P>|z| = 0.01), " I choose clothing items from organic production" (β = 0.39; P>|z| = 0.01) stand out for their positive influence, and the statement "I select clothes that include labels that guarantee the absence of chemical pollutants" (β = -0.47, P>|z| = 0.00) stands out for its negative influence. Complementarily, the selection of fair trade clothes positively affects the frequency of donating or exchanging clothes (β = 0.35, P>|z| = 0.02), and the selection of organic clothing positively affects the frequency of patching or mending clothes (β = 0.31, P>|z| = 0.03).

**Table 5:** Logistic regression of the centennial generation.

	I buy second-hand clothing		I wear patched and mended clothing		I give away or swap unwanted clothing items that I no longer wear		Instead of buying a new piece of clothing for a special occasion, I borrow something		I look for other possible uses of unwanted clothing items		I air my clothing items before deciding whether they need washing		I throw away clothing items that I no longer wear	
	β	P>|z|	β	P>|z|	β	P>|z|	Β	P>|z|	β	P>|z|	β	P>|z|	β	P>|z|
I avoid buying clothing items that originate in countries with poor working conditions	**0.39**	**0.01**	0.15	0.23	-0.09	0.46	0.18	0.16	0.09	0.44	0.12	0.32	0.17	0.20
I choose clothing items from fair-trade production	0.15	0.30	0.10	0.49	**0.35**	**0.02**	0.11	0.41	0.05	0.70	0.22	0.10	-0.14	0.29
I choose clothing items from organic production (e.g., made from organic cotton)	**0.39**	**0.01**	**0.31**	**0.03**	0.09	0.54	0.08	0.61	0.08	0.59	0.22	0.12	0.03	0.86
I select clothing that includes labels guaranteeing the absence of chemical pollutants	**-0.47**	**0.00**	-0.17	0.21	-0.11	0.46	0.05	0.72	-0.00	0.98	-0.05	0.73	-0.01	0.92
I choose high quality and long-lasting clothing items	-0.13	0.22	-0.15	0.17	0.16	0.15	-0.09	0.39	0.02	0.86	0.07	0.51	0.10	0.37
Observations	115		115		115		115		115		115		115	
Prob > chi2	0.00		0.05		0.05		0.15		0.68		0.01		0.62	
Pseudo R2	0.05		0.03		0.03		0.02		0.01		0.03		0.01	

In the case of the older persons, the results presented in Table 6 show that all ologit regressions, except the regression with the dependent variable "I throw away clothing items that I no longer wear", obtained adequate goodness-of-fit at 99% (p <0.01) or 95% (p <0.05) confidence, according to the Chi2 test. In this group, the statement "I avoid buying clothing items that originate in countries with poor working conditions" stands out because it had positive regression coefficients on regressions with dependent variables "I buy second-hand clothing" (β = 0.38, P>|z| = 0.01), "I wear patched and mended clothing" (β = 0.21, P>|z| = 0.09), "instead of buying a new piece of clothing for a special occasion, I borrow something" (β = 0.48, P>|z| = 0.00) and "I look for other possible uses of unwanted clothing items" (β = 0.36, P>|z| = 0.01). Additionally, as in the case of men, negative and significant regression coefficients at 99% (P<0.01), 95% (P<0.05) or 90% (P<0.10) confidence stand out in the sentence "I select garments of high quality in their materials and elaboration, and with long duration", specifically, in the regressions with dependent variables "I buy second-hand clothing" (β = -0.29, P>|z| = 0.01), "I wear patched and mended clothing" (β = -0.17, P>|z| = 0.09), "instead of buying a new piece of clothing for a special occasion, I borrow something" (β = -0.21, P>|z| = 0.06) and "I look for other possible uses of unwanted clothing items" (β = -0.41; P>|z| = 0.00). Complementarily, selecting clothes that are produced under fair trade guidelines affects the dependent variables "I give away or swap unwanted clothing items that I no longer wear" (β = 0.21, P>|z| = 0.05) and "I air my clothing items properly before deciding whether they need washing" (β = 0.20, P>|z| = 0.08); selecting clothes produced organically positively affects the dependent variables "I wear patched and mended clothing" (β = 0.25, P>|z| = 0.07), "instead of buying a new piece of clothing for a special occasion, I borrow something" (β = 0.30, P>|z| = 0.05) and "I look for other possible uses of unwanted clothing items" (β = 0.33, P>|z| = 0.02).

**Table 6:** Logistic regression of older adults.

	I buy second-hand clothing		I wear patched and mended clothing		I give away or swap unwanted clothing items that I no longer wear		Instead of buying a new piece of clothing for a special occasion, I borrow something		I look for other possible uses of unwanted clothing items		I air my clothing items before deciding whether they need washing		I throw away clothing items that I no longer wear	
	β	P>|z|	β	P>|z|	β	P>|z|	β	P>|z|	β	P>|z|	β	P>|z|	β	P>|z|
I avoid buying clothing items that originate in countries with poor working conditions	**0.38**	**0.01**	**0.21**	**0.09**	0.17	0.17	**0.48**	**0.00**	**0.36**	**0.01**	0.14	0.27	0.20	0.13
I choose clothing items from fair-trade production	-0.04	0.75	-0.02	0.87	**0.21**	**0.05**	-0.10	0.45	-0.03	0.82	**0.20**	**0.08**	-0.20	0.08
I choose clothing items from organic production (e.g., made from organic cotton)	0.12	0.43	**0.25**	**0.07**	0.05	0.70	**0.30**	**0.06**	**0.33**	**0.02**	0.04	0.80	0.21	0.13
I select clothing that includes labels guaranteeing the absence of chemical pollutants	-0.01	0.92	0.04	0.77	0.13	0.32	-0.11	0.48	0.13	0.33	0.16	0.22	-0.25	0.06
I choose high quality and long-lasting clothing items	**-0.29**	**0.01**	**-0.17**	**0.09**	0.03	0.76	**-0.21**	**0.06**	**-0.41**	**0.00**	0.03	0.76	0.04	0.69
Observations	125		125		125		125		125		125		125	
Prob > chi2	0.00		0.01		0.00		0.00		0.00		0.00		0.23	
Pseudo R2	0.05		0.04		0.06		0.06		0.09		0.05		0.02	

Mainly, these results indicate that in older persons, selecting clothes manufactured in countries with good working conditions and produced organically positively affects purchase or post-purchase actions that benefit environmental and social sustainability; conversely, selecting products for quality negatively affects sustainable purchase and post-purchase actions, particularly the purchase of secondhand clothes, patching or mending clothes, borrowing clothes and finding new uses for them.

## 6. Discussion

This research demonstrates that consumer valuation of sustainable clothing attributes affects the frequency of sustainable clothing purchase and post-purchase actions. The study proposes the following research questions: Does people’s evaluation of attributes for selecting clothing, included in purchase choices, impact post-purchase actions embedded in sufficient and frugal consumption? Does the impact differ according to people’s gender and age generation? The findings demonstrate that product selection based on sustainable attributes affects sustainable purchasing and post-purchase behaviours, and this effect differs by gender and age generation.

The results of the logistic regressions, supported by hypothesis tests to assess the significance of the coefficients and the goodness of fit of the regression models, reveal that the frequency of clothing selection in terms of its sustainable attributes influences the frequency of sustainable purchase and post-purchase behaviours. The p-values of the regression coefficients, less than 0.10, 0.05 or 0.01, imply that the regression coefficients are significant at the 99%, 95% or 90% confidence level. These findings support Hypothesis 1 of this research.

The ologit regressions also show that this influence is distinct between men and women. The significant regression coefficients show that in the group of women evaluated, the statement "I avoid buying clothing items that originate in countries with poor working conditions." is relevant, as it positively influences behaviours such as the purchase of second-hand clothes, borrowing clothes for special occasions, and finding new uses for clothes and airing clothes before deciding to wash them. In the case of men, the selection of clothing with organic materials is an important attribute that positively influences buying second-hand clothes, patching or mending clothes and airing clothes before deciding to wash them. Conversely, for men, clothing selection by quality demonstrates a significant negative influence on sustainable purchasing and post-purchase behaviours, specifically buying second-hand clothes, patching or mending clothes, borrowing clothes for a special occasion and finding other uses for unwanted clothing items. This evidence allows us to validate Hypothesis 2 of this research, which states that the valuation of sustainable attributes of clothing differentially affects the frequency of sustainable purchase and post-purchase consumption behaviours between men and women.

The ologit regressions also show that this influence is distinct between the centennial generation and older persons. Regarding the centennial generation, the results mainly show that sustainable attributes for clothing selection affect the frequency of secondhand clothing purchases. Particularly, in the centennial generation, avoiding selecting clothes that originate in countries with poor labour conditions and selecting clothes produced organically positively influences the purchase of secondhand clothes; conversely, selecting clothes that guarantee the absence of pollutants negatively influences the purchase of secondhand clothes. In the case of older persons, the clothing selection attributes "I avoid buying clothing items that originate in countries with poor working conditions" and "I choose clothing items from organic production" stand out, as they have a positive influence on buying secondhand clothes, patching or mending clothes and borrowing clothes for special occasions. Additionally, evidence that the selection of clothing for quality negatively affects buying second-hand clothes, patching or mending clothes, borrowing clothes for special occasions, and finding other uses for unwanted clothing items is relevant for older persons. This evidence allows us to validate Hypothesis 3 of this research, which states that the valuation of sustainable attributes of clothing differentially affects the frequency of sustainable purchase and post-purchase behaviours between the centennial generation and older people.

With the theoretical support from response hierarchy models [36, 38], this research shows that clothing preference based on attributes such as organic production, fair-trade and pollutant-free sales, and quality influences subsequent purchase and post-purchase behaviour, which is consistent with these models. The findings are also coherent with previous research supporting differences in sustainability concerns, sustainable product selection and sustainable purchase and post-purchase actions by gender [68, 69]. They are also consistent with previous publications that recognise differences in sustainability concerns, sustainable product selection and sustainable purchase and post-purchase actions by age generation [25, 75].

This work is a new contribution to knowledge about the attributes of sustainable clothing that affect purchase and post-purchase behaviours, distinguishing this influence according to gender and age generation. The findings are involved in relevant and widely studied sustainable clothing behaviours, such as buying secondhand clothing and repairing, reusing and donating clothes. A better understanding of sustainable apparel attributes that favour or disfavour the frequency of sustainable purchasing and post-purchase actions is of high importance for sustainable clothing consumption due to the enormous volume of waste generated by the disposal of apparel and the poor working conditions in countries with major fast-fashion production, such as Bangladesh, Indonesia, China, Turkey and Vietnam [91]. The New York Times published the concept “Fast Fashion” to describe Zara’s goal of taking only 15 days from the design of a garment to its sale in shops [92]. Lax environmental regulations in these countries allow companies to mass-produce clothing without legal protest, causing environmental poverty through a volatile combination of water, chemicals and waste [93].

The results of this study have practical utility for governmental and nongovernmental organisations concerned with sustainable clothing consumption. Knowledge of the attributes of clothing selection that positively or negatively affect secondhand purchasing, and actions related to fashion clothing disposal behaviour should guide policies and regulations to inform the characteristics of these products with higher detail and prominence. Governments could mandate the incorporation of labels or seals that more clearly report conditions on clothing, such as the use of organic materials, the absence of pollutants and fair-trade production, in a similar way to the seals on food packaging that warn of high sugar and saturated fat components. Previous studies have argued that increased awareness of the social and environmental impact of clothing contributes to changing consumers’ purchasing, consumption and disposal behaviours [94]; hence, governmental and nongovernmental organisations should develop communication campaigns highlighting the importance of such sustainable attributes in products and regulate the wholesale and retail distribution of clothing.

Information from this research can also guide the commercial strategies of apparel companies seeking to capture the preference of market segments that value sustainable products. Because some attributes of clothing selection positively influence clothing donation, exchange or repair, fashion companies could offer benefits such as discounts on new clothing by receiving used clothing in part payment and offering repair and maintenance services for clothing already purchased. They could also connect these consumers with organisations, communities or other clients looking for used clothing or train consumers to transform their branded clothing for new uses.

Additionally, the results indicate that a preference for quality discourages sustainable purchase and post-purchase actions among men and older persons. These negative relationships between the preference for quality and durability and the purchase of secondhand clothing are consistent with the study by Eike et al. [95], who found that people discard clothing that they perceive to be of poor quality or poor fit. However, in the group of young people, no significant and negative regression coefficients were found between the search for quality and sustainable purchase and post-purchase behaviours, and in the women group, only one significant negative regression coefficient was found. In other words, the results suggest that the centennial generation and women show fewer objections to preferring higher quality and more durable clothing—according to their perception—and to buying secondhand clothing and patching and borrowing garments.

One explanation for this difference is that quality may be perceived differently among age groups and genders. Quality is defined as the consumer’s overall assessment of how well goods or services perform [96] and as the superiority of a product when compared to an alternative product from a market perspective [97]. These definitions suggest that quality is a general concept that depends on personal interpretation of a product. In this sense, younger people and women might relate quality mostly to slow fashion attributes such as longer production times, use of local materials, and sustainability. In contrast, older people and men may have a different perspective on quality, for example, related to exclusivity, brand tradition and sophistication; these characteristics are more closely related to luxury or premium product attributes [98, 99]. Therefore, the evidence of this research suggests that public and private organisations concerned with sustainability must inform older people and men that sustainability is currently an important characteristic of quality; likewise, to further promote sustainable purchasing and post-purchase behaviours among men and older persons who are quality seekers and who associate quality with fast fashion features.

## 7. Conclusions

This research provides new evidence about the importance of clothing attributes on the frequency of sustainable purchase and post-purchase actions, such as secondhand purchase, repair, exchange, donation and ventilation of clothing, distinguishing results by gender and age generation. The results show that for women, the manufacture of clothing in countries with good working conditions positively influences their sustainable purchasing and post-purchase behaviours. In the case of the centennial generation, the attributes for clothing selection mostly positively affect the purchase of secondhand clothing. In the group of men and older persons, it is remarkable that the evidence found supports the negative effect of clothing selection by quality on sustainable purchase and post-purchase behaviours. Additionally, this research recognises a higher frequency of sustainable purchase and post-purchase actions incorporated in the sufficient and frugal consumption factor in women and centennial generation and a higher valuation of quality, incorporated in the purchase choices factor, in men and older persons.

## 8. Limitations and future research

Future research should deepen the understanding of the influence of clothing attributes on sustainable purchase and post-purchase behaviour by using qualitative techniques to obtain more meaning from the results of this research. Precisely one of the limitations of studies with a quantitative approach is the absence of motives, reasons or meanings in the results that can be found mostly in qualitative studies. In addition, this research was conducted on university students in Chile who were mostly workers enrolled in executive programmes and they have the sociodemographic conditions described in the sample section. The characteristics of the sample are a limitation for the generalisability of the results; consequently, it is pertinent to conduct further research, identifying the results by gender and age generation, in countries with different cultures in Asia, Europe and Africa. Finally, future studies should further study the discrepancies between quality and sustainable consumption among men and older persons using qualitative techniques such as interviews and focus groups to better understand the reasons and emotions that generate differences.
